# Selective Spectrofluorimetric Approach for the Assessment of Two Antipsychotic Drugs through Derivatization with *O*-Phthalaldehyde

**DOI:** 10.3390/ph15101174

**Published:** 2022-09-22

**Authors:** Hassan M. A. Hassan, Ibrahim H. Alsohaimi, Mohamed Y. El-Sayed, Ismail I. Ali, Akram M. El-Didamony, Hamud A. Altaleb, Mutairah S. Alshammari

**Affiliations:** 1 Chemistry Department, College of Science, Jouf University, Sakaka 2014, Saudi Arabia; 2 Chemistry Department, Faculty of Science, Zagazig University, Zagazig 44519, Egypt; 3 Department of Chemistry, Faculty of Science, Islamic University of Madinah, Madinah 41477, Saudi Arabia

**Keywords:** spectrofluorimetry, derivatization reaction, *o*-phthalaldehyde, aripiperazole, clozapine

## Abstract

A facile and sensible spectrofluorimetric approach for the measurement of two antipsychotic medications, aripiperazole (ARP) and clozapine (CLZ), was devised and validated. The approach involves reacting the examined medicines with *o*-phthalaldehyde in the presence of β-mercaptoethanol in a borate buffer of pH 9.0 and pH 10 for ARP and CLZ, respectively, to produce a robustly fluorescent compound that is detected at 450 nm following excitation at 340 nm. The experimental variables influencing the performance and product stability were thoroughly investigated and optimized. Under optimal conditions, the intensity of the fluorescence was linear during a concentration range of 0.1–0.5 μg/mL, with a limit of detection (0.0391 and 0.0400 μg/mL) and limit of quantitation (0.1035 and 0.1332 μg/mL), respectively, for ARP and CLZ. The suggested approach was successful in analyzing commercialized tablets. A statistical investigation of the results produced by the suggested and standard methods showed no remarkable variation in the precision and accuracy of the two approaches. A chemical mechanism using *o*-phthalaldehyde was proposed.

## 1. Introduction

Schizophrenia is a serious psychiatric condition that influences around 1.5 percent of the world’s community and treated mostly with antipsychotic medicines. Indeed, antipsychotic drugs are one of the pharmaceutical industry products experiencing rapidly increasing demand [1]. Aripiprazole (ARP) and clozapine (CLZ) are two antipsychotics. When compared to traditional antipsychotics, they have been shown to be effective in treating symptoms of schizophrenia and be minimally prone to elicit extrapyramidal side effects. However, large doses of these atypical antipsychotics are thought to raise the likelihood of extrapyramidal or other adverse effects [2,3,4,5,6,7].

Aripiprazole (ARP) is a benzisoxazole antipsychotic drug of the sixth generation. According to a review of the literature, chromatographic methods [8,9,10,11,12,13,14,15], electrophoresis [16], and spectrophotometric methods [17,18] are among the few analytical techniques for detecting aripiprazole in drug formulations and biological materials.

Clozapine (CLZ) also belongs to a kind of diazepine and there is no mention of the medication in any pharmacopoeia. Various techniques for clozapine assessment were reported. Liquid chromatography [19,20,21,22], HPLC [23,24,25,26], GC [27], electrophoresis [28,29], voltammetry [30,31,32], and spectrophotometric [33,34,35] are among these approaches. The majority of these solutions have some downsides. HPLC and other approaches require costly instruments that were not readily applicable in the majority of laboratories, and the operations were not easy to carry out. As a result, we set out to create a simple, sensitive, and selective spectrofluorimetric approach for determining the antipsychotic medicines aripiprazole and clozapine by derivatization with *o*-phthalaldehyde.

## 2. Results and Discussions

**Figure 1:**
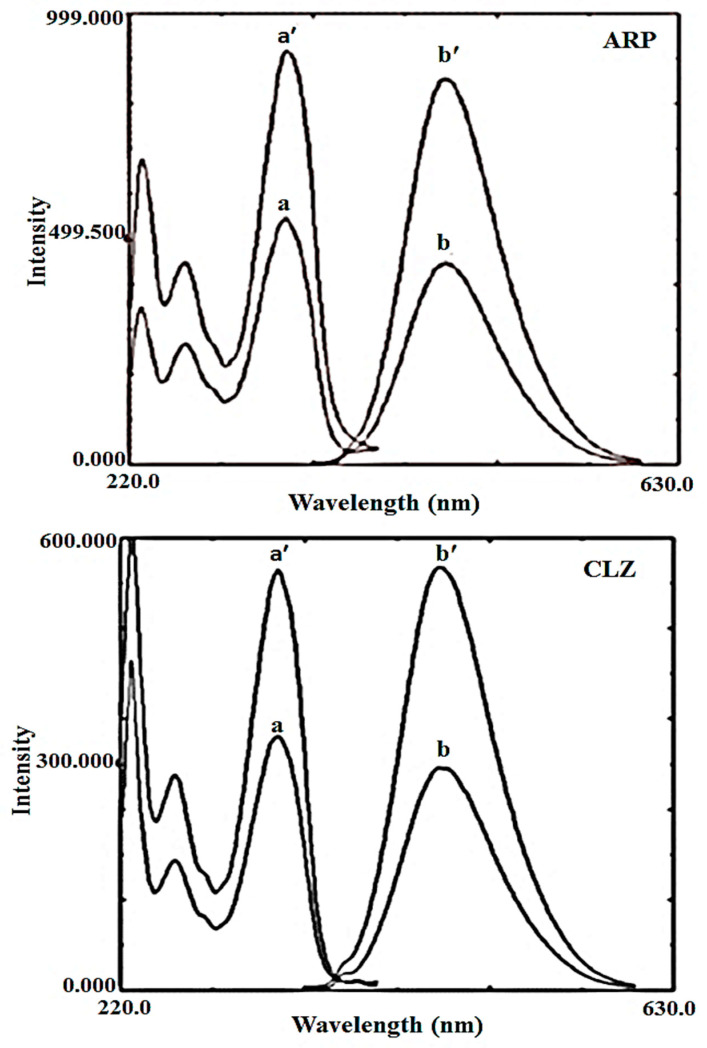
Excitation (a, b) and emission spectra (a′, b′) for the blank of *o*-phthalaldehyde and sample (reaction product of ARP and CLZ with *o*-phthalaldehyde in borate buffer), respectively.

As a luminous derivatizing reagent for amino-containing compounds, *o*-phthalaldehyde in conjunction with a mercapto molecule, such as β-mercaptoethanol, is commonly used. Several medicinal molecules have been identified using this method [36,37,38,39,40]. In the presence of β-mercaptoethanol in a borate buffer, ARP and CLZ reacted with benzene-1,2-dicarboxaldehyde to generate a strongly fluorescent compound with maximal fluorescence at 450 nm following excitation at 340 nm (Figure 1).

### 2.1. Optimization of Experimental Conditions

**Scheme 1:**
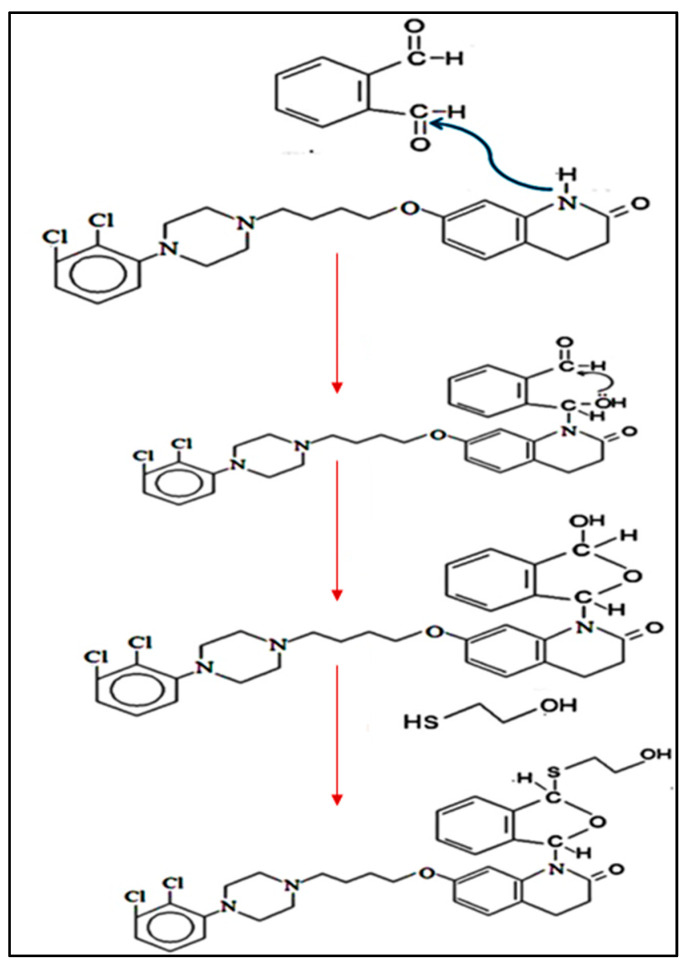
Proposed pathway of the reaction between ARP and *o*-phthalaldehyde in the presence of β-mercaptoethanol in a borate buffer.

The condensation process between ARP and CLZ with *o*-phthalaldehyde took place at pH 9 and pH 10, in the presence of β-mercaptoethanol, which maintained the product. Scheme 1 depicts the suggested mechanism for ARP as an example. The fluorescence characteristics of the formed product, and the various experimental parameters influencing the formation of the product and product durability have been thoroughly examined and maximized. These variables have been altered separately, while the remains remained stable. The variables comprising concentration of the reagents, time, and pH were all considered.

#### 2.1.1. Impact of pH

**Figure 2:**
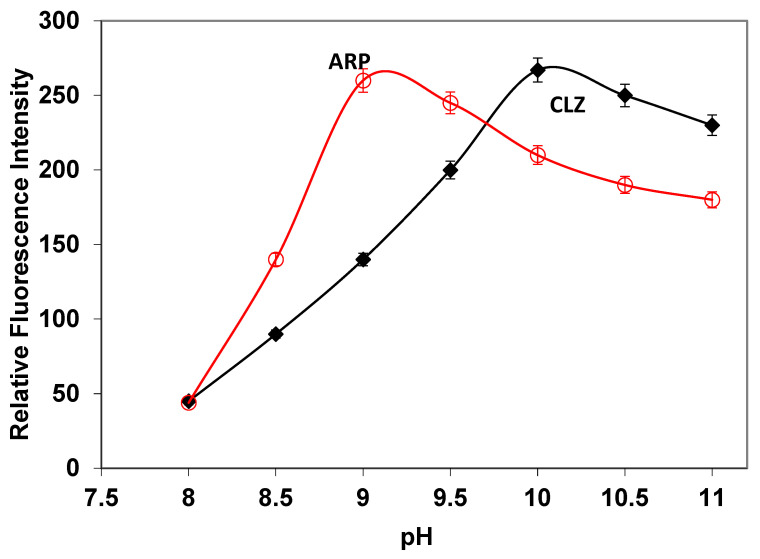
Effect of pH of borate buffer on the relative fluorescence intensity of the reaction product of ARP and CLZ with *o*-phthalaldehyde.

The pH influence of three various buffers was investigated to optimize the reaction parameters. At respective alkaline operating ranges, bicarbonate, phosphate, and borate buffers have been used. Buffers containing amino moieties, such as triethylamine, were avoided because they interact with *o*-phthalaldehyde. For 45 min, the reaction was performed at ambient temperature. The ultimate intensity of the fluorescence was attained for ARP and CLZ utilizing borate buffer at pH 9 and 10, respectively. After which, the intensity dropped due to excessive background intensity (Figure 2). Furthermore, a test of the buffer volume revealed that reaction mixtures were accomplished to 10 mL with borate buffer.

#### 2.1.2. Influence of *O*-Phthalaldehyde Volume

**Figure 3:**
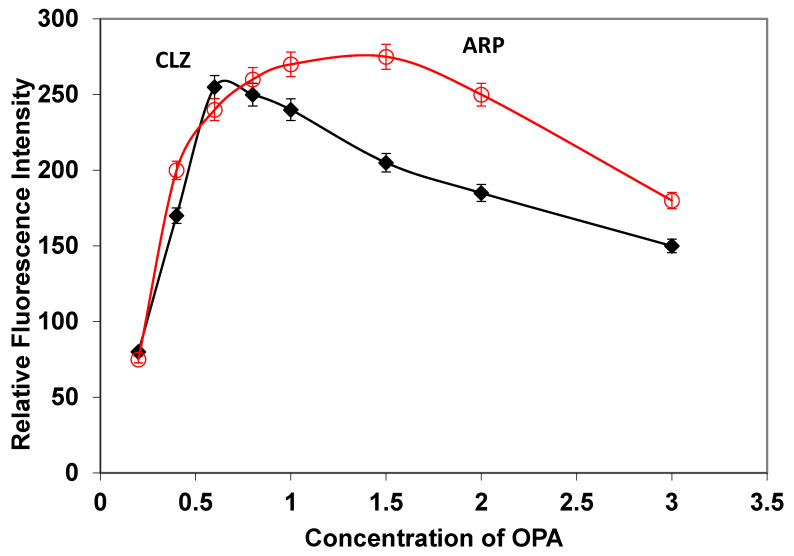
Effect of concentration of *o*-phthalaldehyde, (0.1% *w*/*v*) on the fluorescence intensity of the reaction product of ARP and CLZ in borate buffer.

The effect of *o*-phthalaldehyde volume was investigated by introducing varied volumes of 0.1% (*w*/*v*) reagent solution. The volumes were varied from 0.2 to 3.0 mL. Maximum intensities were observed to be at 1.5 and 0.6 mL with ARP and CLZ, respectively (Figure 3), after which the intensity gradually decreased.

#### 2.1.3. Influence of β-Mercaptoethanol Volume

**Figure 4:**
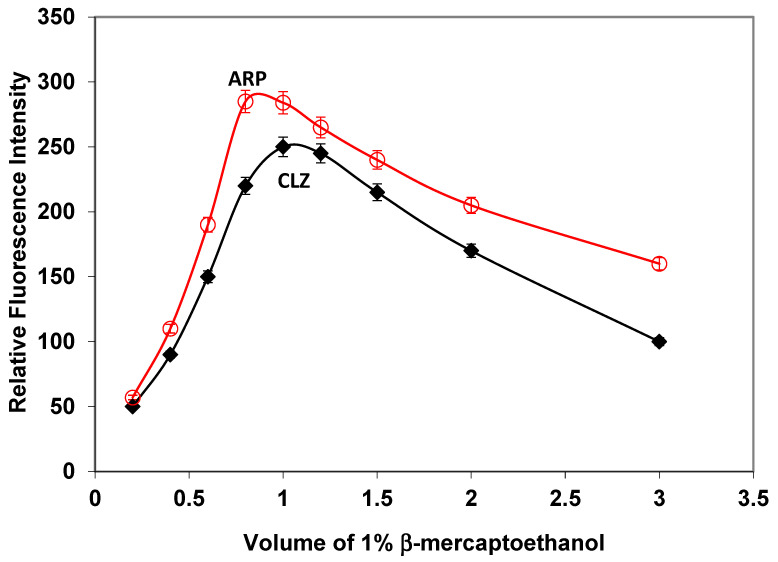
Effect of volume of β-mercaptoethanol (1.0% *v*/*v*) on the fluorescence intensity of the reaction product of ARP and CLZ with *o*-phthalaldehyde in borate buffer.

The effect of volume on β-mercaptoethanol was investigated using varied quantities of 1.0% (*v*/*v*) reagent solution. It was shown that developing the reagent volume results in a corresponding rise in the intensity of the fluorescence of the product up to 1.0 mL (Figure 4). However, volume more than 1.0 mL caused a decline in fluorescence. As a result, 1.0 mL of 1.0% β-mercaptoethanol solution was selected as the best reagent volume. To stabilize the reaction product of ARP and CLZ with *o*-phthalaldehyde, β-mercaptoethanol was added.

#### 2.1.4. Influence of Reaction Time

**Figure 5:**
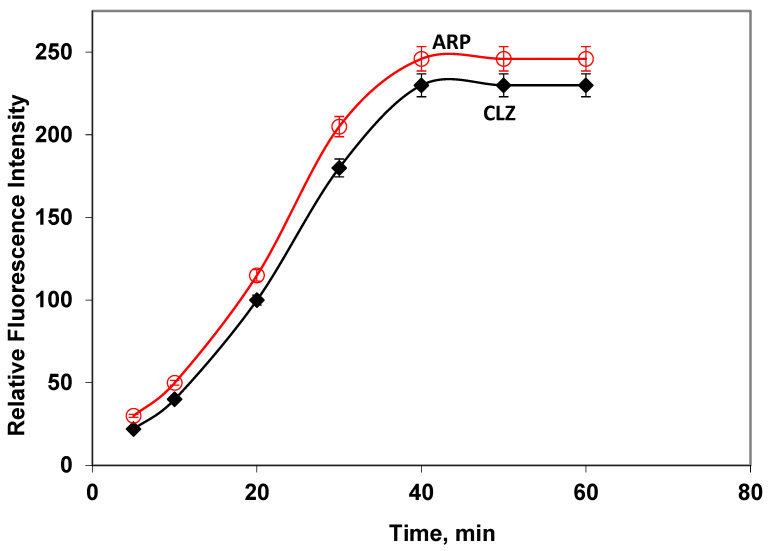
Effect of reaction time on the relative fluorescence intensity of the reaction product of ARP and CLZ with *o*-phthalaldehyde.

The required time for a successful derivatization process was investigated. It is worth noting that, raising the process time led to a progressive raising in the intensity of the fluorescence of the product up to 40 min, after that it remained unchanged. As a result, leaving the reaction mixture to settle for 40 ± 5 min is sufficient for the greatest intensity (Figure 5).

#### 2.1.5. Impact of Temperature

The impact of various different temperatures with a constant heating time was studied. Notably, raising the temperature caused a steady decline in the intensity of the fluorescence of the product obtained. As a consequence, the process was carried out at ambient temperature. In terms of durability, the generated compound was shown to be durable over a 2 h period.

### 2.2. Linearity and Range

**Figure 6:**
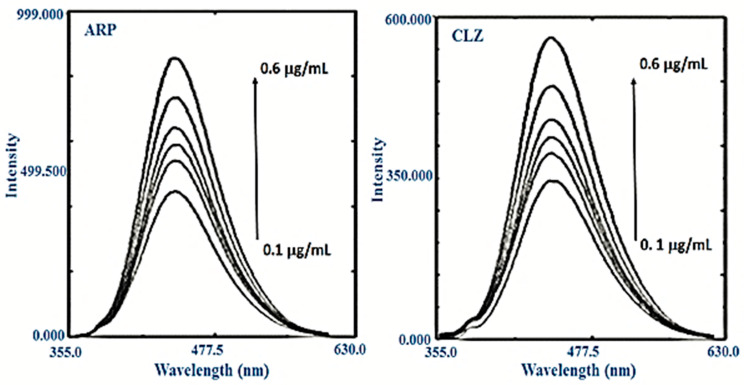
Emission spectra of *o*-phthalaldehyde with increasing ARP and CLZ concentrations (0.1–0.06 μg/mL) in buffer borate.

The regression plot revealed a linear correlation of intensity of the fluorescence on the drug concentration over the spectrum shown in Figure 6. The following equations resulted from a linear regression assessment of the data:

F = −21 + 664C (R = 0.9989) for ARP
(1)

F = −15.9 + 521C (R = 0.9983) for CLZ
(2)

where F denotes the intensity, R the regression coefficient, and C the drug concentration (μg/mL).

### 2.3. Detection Limit

The proposed method was validated according to the International Conference on Harmonization (ICH) Guidance Documents testing [41]. The limit of detection (LOD) (0.0391 and 0.0400 μg/mL) and limit of quantitation (LOQ) (0.1035 and 0.1332 μg/mL) were determined for ARP and CLZ, respectively (Table 1).

### 2.4. Accuracy and Precision

The suggested approach’s precision was estimated on the basis of intermediate precision (intra-day and inter-day). Three varied drug concentrations were examined in five replicates on the same day (intra-day precision) and for seven days in a row (inter-day precision). Table 2 and Table 3 describe the analytical findings from the examination. The accuracy was reported as a relative error percentage (Er %), and intra-day and inter-day reliability were estimated as relative standard deviations (RSD %). The suggested approach’s level of precision was appropriate for the quality assurance assessment of the drugs under consideration.

### 2.5. Tablets Analysis

The suggested approach was utilized to measure the drug content in the tablets. The acquired findings obtained were sufficiently precise and accurate, as evidenced by the good recovery percentage and the RSD % below 4.5% (Table 4).

## 3. Materials and Methods

### 3.1. Instrumentation

Fluorescence intensities were measured using a Shimadzu RF-5301 PC spectrofluorimeter (Kyoto, Japan) outfitted with a xenon lamp (150 W) and 1 cm quartz cells. Slits of 5 mm were used to fix the excitation and emission monochromators. For heating, an electrical thermostatic water bath with a temperature range of 35–100 °C was utilized. The pH values of the buffer solutions were measured using the Jenway instrument pH-meter (combined electrode).

### 3.2. Reagents Preparation

All of the reagents utilized were of analytical grade and applied as obtained.

- Stock solutions preparation: Pharmaceutical grade ARP and CLZ confirmed to be 99.85% pure was obtained as a gift, kindly supplied from the Egyptian International Pharmaceutical Industries Company (EIPICo), Egypt. Separate pure ARP and CLZ stock solutions were developed by dissolution 10 mg of each medication in concentrated H_2_SO_4_ (1.0 mL) followed by completion to 100 mL of purified water.
- *o*-phthalaldehyde was obtained from Sigma (St. Louis, MO, USA). A 0.1% (*w*/*v*) methanol stock solution was freshly prepared.
- Sigma also supplied β-mercaptoethanol (St. Louis, MO, USA). A 0.1% (*v*/*v*) ethanol stock solution was also prepared.
- Calculated amounts of 0.2 M boric acid and 0.2 M NaOH were used to prepare a borate buffer solution to adjust the pH to 9.0, 10 with a pH meter.

### 3.3. Recommendations for General Processes

ARP and CLZ solution with drug concentrations of 0.1–0.5 μg/mL were added to a set of 10 mL volumetric flasks, followed by 1.5 mL and 0.6 mL of 0.1% (*w*/*v*) *o*-phthalaldehyde for both ARP and CLZ, respectively. Afterward, 0.8 mL and 1.0 mL of β-mercaptoethanol 1.0% (*v*/*v*) was introduced to each flask and thoroughly mixed. The reaction mixtures were kept at ambient temperature for 45 min before the volumes were completed to the mark using pH 9 and pH 10 borate buffers for ARP and CLZ, respectively. The fluorescence of the obtained solutions was estimated at 450 nm following excitation at 340 nm in comparison to a blank solution made in the same way, except the drugs’ addition. The calibration curve was created by plotting the fluorescence intensity against the final medication concentrations (μg/mL).

### 3.4. Tablet Procedure

At least 10 tablets of the medications were ground and well combined in a small dish. Then, 10 mg ARP and CLZ were solubilized with conc. H_2_SO_4_ (1.0 mL) in distilled water, followed by filtration into a volumetric flask (100 mL), and then diluted with water. Various concentrations were prepared using the obtained stock solution. The actual concentration of the tablets was assessed by a calibration curve.

## 4. Conclusions

A novel, facile and selective spectrofluorimetric method for determining ARP and CLZ has been successfully developed. The interaction of ARP and CLZ with *o*-phthalaldehyde in the presence of β-mercaptoethanol resulted in the formation of a luminous derivative. The approach was successfully used to assess ARP and CLZ in their pure state and in tablets with no interference from additives. As a result, the suggested method may be appropriate for routine investigation of drugs in quality control laboratories.

## Figures and Tables

**Table 1:** Analytical parameters for spectrofluorometric assessment of aripiprazole and clozapine.

Parameters	ARP	CLZ
Linear range, μg/mL	0.1–0.5	0.1–0.5
Correlation coefficient (r)	0.9989	0.9983
Linear regression equation		
S_y/x_	4.049	3.877
Intercept (a)	−21	−15.9
Slope (b)	664	521
S.D. of slope (S_b_)	12.806	12.261
S.D. of intercept (S_a_)	7.014	6.715
LOD, μg/mL	0.0391	0.0400
LOQ, μg/mL	0.1035	0.1332

**Table 2:** Evaluation of intra-day accuracy and precision of the proposed spectrofluorimetric method.

Drug	Drug Taken, μg/mL	Recovery ^a^, %	RSD,%	RE ^b^, %	SE
ARP	0.2	99.994	1.75	−0.004	3.71
	0.3	99.997	1.53	−0.006	4.81
	0.4	99.989	1.67	−0.004	3.97
CLZ	0.2	99.968	2.21	−0.004	3.75
	0.3	99.989	1.49	−0.007	3.04
	0.4	99.992	1.49	−0.004	3.29

^a^ Mean value of five determinations. ^b^ RE: Relative error.

**Table 3:** Evaluation of inter-day accuracy and precision of the proposed spectrofluorimetric method.

Drug	Drug Taken, μg/mL	Recovery ^a^, %	RSD, %	RE ^b^, %	SE
ARP	0.2	99.996	2.71	−0.004	4.37
	0.3	99.998	2.21	−0.002	4.14
	0.4	99.994	2.12	−0.006	4.28
CLZ	0.2	99.998	2.28	−0.002	3.39
	0.3	99.996	2.28	−0.004	3.72
	0.4	99.994	2.45	−0.006	4.08

^a^ Mean value of five determinations. ^b^ RE: Relative error.

**Table 4:** Recovery of the studied drugs from their pharmaceuticals using the proposed spectrofluorometric method.

Drug	Drug Formulation	Drug Taken μg/mL	Drug Found, μg/mL	Recovery ^a^ %	RSD, %	RE ^b^, %
ARP	Aripiprex ^c^, 10 mg/tab	0.2	0.206	103	4.45	3.03
		0.3	0.303	101	3.79	1.31
		0.4	0.408	102	3.74	2.07
CLZ	Clozapex ^d^, 100 mg/tab	0.2	0.199	99.9	2.44	−0.05
		0.3	0.299	99.9	2.75	−0.05
		0.4	0.395	98.9	2.37	−1.06

^a^ Mean value of five determinations. ^b^ RE: Relative error. ^c^ S.P.I. for Al Andalous Medical Company, 6th of October City, Egypt. ^d^ APEX Pharma S.A.E.,10th of Ramadan City, Egypt.

## Data Availability

Data is contained within the article.
